# Supplementary material for: Enhancing intracellular accumulation and target engagement of PROTACs with reversible covalent chemistry
Source: Nat Commun. 2020 Aug 26;11:4268. doi: 10.1038/s41467-020-17997-6 (PMC7450057; doi:10.1038/s41467-020-17997-6)
Supplement: Supplementary file 3 — Description of Additional Supplementary Files [file 41467_2020_17997_MOESM3_ESM.docx]

**Description of Additional Supplementary Files**

**Title: Supplementary Data 1.**

Description: The raw data and curves of BTK binding assay

**Title: Supplementary Data 2.**

Description: The raw data of BTK degraders proteomics

**Title: Supplementary Data 3.**

Description: The materials, procedure and bioanalytical method of permeability determination using Lipid-PAMPA method

**Title: Supplementary Data 4.**

Description: The raw data of quantification of intracellular concentration of RC-1, IRC-1 and RNC-1 by LC-MS
