# Supplementary material for: Enhancing intracellular accumulation and target engagement of PROTACs with reversible covalent chemistry
Source: Nat Commun. 2020 Aug 26;11:4268. doi: 10.1038/s41467-020-17997-6 (PMC7450057; doi:10.1038/s41467-020-17997-6)
Supplement: Supplementary file 8 — Reporting Summary [file 41467_2020_17997_MOESM8_ESM.pdf]

natureresearch

Corresponding author(s): Jin Wang

Last updated by author(s): Jul 12, 2020

Reporting Summary

Nature Research wishes to improve the reproducibility of the work that we publish. This form provides structure for consistency and transparency in reporting. For further information on Nature Research policies, see Authors & Referees and the Editorial Policy Checklist .

Statistics

For all statistical analyses, confirm that the following items are present in the figure legend, table legend, main text, or Methods section.

n/a	Confirmed	
☐	☒	The exact sample size (*n*) for each experimental group/condition, given as a discrete number and unit of measurement
☒	☐	A statement on whether measurements were taken from distinct samples or whether the same sample was measured repeatedly
☐	☒	The statistical test(s) used AND whether they are one- or two-sided<br>*Only common tests should be described solely by name; describe more complex techniques in the Methods section.*
☒	☐	A description of all covariates tested
☒	☐	A description of any assumptions or corrections, such as tests of normality and adjustment for multiple comparisons
☐	☒	A full description of the statistical parameters including central tendency (e.g. means) or other basic estimates (e.g. regression coefficient) AND variation (e.g. standard deviation) or associated estimates of uncertainty (e.g. confidence intervals)
☐	☒	For null hypothesis testing, the test statistic (e.g. *F*, *t*, *r*) with confidence intervals, effect sizes, degrees of freedom and *P* value noted<br>*Give P values as exact values whenever suitable.*
☒	☐	For Bayesian analysis, information on the choice of priors and Markov chain Monte Carlo settings
☒	☐	For hierarchical and complex designs, identification of the appropriate level for tests and full reporting of outcomes
☒	☐	Estimates of effect sizes (e.g. Cohen's *d*, Pearson's *r*), indicating how they were calculated

Our web collection on statistics for biologists contains articles on many of the points above.

Software and code

Policy information about availability of computer code

Data collection	Biotek Gen 5; GROMACS-2019.2;
Data analysis	Graphpad Prism 8; Microsoft Excel 365; PyMol v2.4.0; ImageJ(NIH);

For manuscripts utilizing custom algorithms or software that are central to the research but not yet described in published literature, software must be made available to editors/reviewers. We strongly encourage code deposition in a community repository (e.g. GitHub). See the Nature Research guidelines for submitting code & software for further information.

Data

Policy information about availability of data

All manuscripts must include a data availability statement. This statement should provide the following information, where applicable:

- Accession codes, unique identifiers, or web links for publicly available datasets
- A list of figures that have associated raw data
- A description of any restrictions on data availability

Supplementary methods and materials, figures and tables are available. All mass spectrometry raw files been deposited in the MassIVE repository housed at UCSD (https://massive.ucsd.edu/) with the accession number MSV000084902. The source data underlying all supplementary figures and tables are provided as the Source Data file. All other data are available from the corresponding authors on reasonable request.

Field-specific reporting

Please select the one below that is the best fit for your research. If you are not sure, read the appropriate sections before making your selection.

Life sciences study design

All studies must disclose on these points even when the disclosure is negative.

Sample size	Western blots are usually ran with duplicates. NanoBRET target engagement assays are performed with triplicates. Cell viability assays were performed with at least triplicates. Based on literature and previous studies, these sample sizes are sufficient.
Data exclusions	No data exclusion
Replication	All the experimental findings have been reproduced.
Randomization	Randomization is irrelevant to this study.
Blinding	No bliding

Reporting for specific materials, systems and methods

We require information from authors about some types of materials, experimental systems and methods used in many studies. Here, indicate whether each material, system or method listed is relevant to your study. If you are not sure if a list item applies to your research, read the appropriate section before selecting a response.

Materials & experimental systems

n/a	Involved in the study	
☐	☒	Antibodies
☐	☒	Eukaryotic cell lines
☒	☐	Palaeontology
☐	☒	Animals and other organisms
☒	☐	Human research participants
☒	☐	Clinical data

Methods

n/a	Involved in the study	
☒	☐	ChIP-seq
☒	☐	Flow cytometry
☒	☐	MRI-based neuroimaging

Antibodies

Antibodies used	anti-BTK (Cell Signaling Technology Cat. No. 8547); anti-CRBN (Cell Signaling Technology Cat. No. 71810); anti-p-BTK (Y223) (Cell Signaling Technology Cat. No. 5082); anti-IKZF1 (Cell Signaling Technology Cat. No. 14859); anti-IKZF3 (Cell Signaling Technology Cat. No. 15103); anti-GAPDH (Cell Signaling Technology Cat. No. 2118); anti-β-actin (Cell Signaling Technology Cat. No. 4970); the HRP-conjugated secondary antibodies (BIO-RAD, Cat. No. 1706515)
Validation	All the antibodies used in this study are commercially and have been validated by their manufacturers. This infromation is available on their website.

Eukaryotic cell lines

Policy information about cell lines

Cell line source(s)	MOLM-14 cell line was obtained from Dr. Conneely at Baylor College of Medicine; the Mino cells were purchased from the American Type Culture Collection (ATCC, Cat. No. CRL-3000); the Wild-type and C481S BTK XLA cell line were gifts from Dr. Woyach at The Ohio State University. HEK 293T/17 cells were purchased from ATCC (Cat. No. CRL-11268); the B cell lymphoma cell line derived from Eu-Myc mice was a gift from Dr. Yulin Li at Houston Methodist Research Institute.
Authentication	None of the cell lines used were authenticated.
Mycoplasma contamination	The cell lines were not tested for mycoplasma contamination.
Commonly misidentified lines (See ICLAC register)	N/A

Animals and other organisms

Policy information about studies involving animals; ARRIVE guidelines recommended for reporting animal research

Laboratory animals	Female ICR mice (weighing 22-28 g) were obtained from the Center for Comparative Medicine of Baylor College of Medicine. Mice were housed 2-4 per cage in an American Animal Association Laboratory Animal Care accredited facility and maintained under standard conditions of temperature (22 ± 2°C), relative humidity (50%) and light and dark cycle (12/12 hours), and had access to food and water ad libitum. Mice were allowed to acclimate to their environment for one week before experiment.

Wild animals	This study did not involve wild animals.
Field-collected samples	This study did not invovle field-collected samples.
Ethics oversight	We have complied with all relevant ethical regulations for animal testing and research. All the animal experiments were approved by the Institutional Animal Care and Use Committee (IACUC) at Baylor College of Medicine.

Note that full information on the approval of the study protocol must also be provided in the manuscript.
